# Floating knees: variables affecting bone union and functional outcomes: a retrospective study of 72 cases

**DOI:** 10.1051/sicotj/2026033

**Published:** 2026-06-17

**Authors:** Yasmine Salese, Matthieu Cotte, Thibault Druel, Frederic Rongieras, Antoine Bertani, Anthony Viste, Bertrand Boyer

**Affiliations:** 1 Université de Lyon, Université Claude Bernard Lyon 1, Université Gustave Eiffel, IFSTTAR, LBMC UMR_T9406 Bron France; 2 Department of Orthopaedic and Trauma Surgery, Hôpital Edouard Herriot, Hospices Civils de Lyon Lyon France; 3 Department of Orthopaedic and Trauma Surgery, Hôpital Lyon Sud, Hospices Civils de Lyon Pierre-Bénite France

**Keywords:** Floating knee, Bone union, Nonunion, Meniscoligamentous injury, Complications, Functional outcomes

## Abstract

*Introduction*: Floating knee involves concomitant and ipsilateral fractures of the femur and tibia, sometimes associated with a patellar fracture. Complications are common, and functional outcomes are often poor. This study aimed to evaluate bone healing and to identify factors influencing nonunion and functional results. *Methods*: This retrospective study included 72 knees, with a mean follow-up of 4.5 years. The primary outcome was bone union. Neurological, vascular and meniscoligamentous injuries were analyzed. Follow-up parameters were clinical, radiological and functional, including the Karlström and Olerud score. *Results*: At least one fracture was open in 69% of cases. Mean time to union was 10.5 months for the femur, 7.5 months for the tibia, and 4.4 months for the patella. Nonunion occurred in 68% of femoral fractures, 57% of tibial fractures, and 42% of patellar fractures. Open fractures and tibial vascular injuries were significant predictors of nonunion. Meniscoligamentous injuries were diagnosed in 32% of cases and were more frequent with increasing Ran stage (*p* = 0.004). Surgical site infection occurred in 30.6% of cases and was significantly associated with open fractures (*p* < 0.001). Functional outcomes worsened with increasing Ran stage, meniscoligamentous injuries, open fractures, and infection. *Conclusion*: Floating knee injuries are characterized by prolonged healing, high rates of nonunion and infection, and limited functional outcomes.

## Introduction

Concomitant ipsilateral fractures of the femur and tibia define the floating knee, a rare but severe injury, typically occurring in the context of high-energy polytrauma. These injuries are associated with substantial morbidity, with reported mortality rates ranging from 5% to 15% and immediate amputation rates reaching up to 25% [1]. In addition to life-threatening complications, they are characterized by high rates of fracture-related complications, with reported nonunion rates ranging from 4% to 28% for the femur and from 3% to 36% for the tibia [2–6], as well as infection rates up to 20–30% in some series [5, 7, 8].

**Figure 1:**
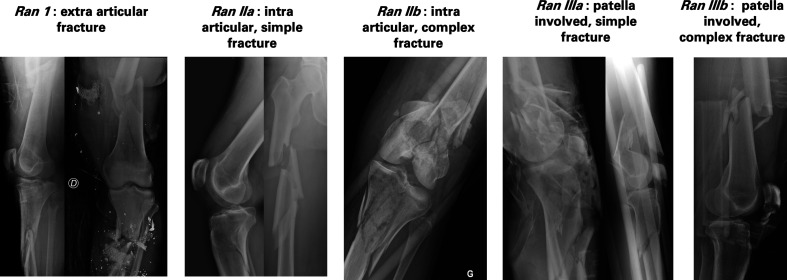
Classification of floating knees by Ran.

Several classification systems have been proposed to describe floating knee injuries, reflecting fracture location and complexity. The most widely used classification by Fraser focuses on diaphyseal and articular involvement, whereas more recent systems, such as the Ran classification, incorporate articular extension and patellar fractures (Figure 1) [9]. Further refinements have been proposed to include soft-tissue and meniscoligamentous lesions, highlighting their prognostic importance [2]. Increasing fracture complexity has consistently been associated with higher rates of complications, including nonunion, infection, stiffness and post-traumatic osteoarthritis [9–12].

In addition to bony injuries, associated soft-tissue and meniscoligamentous lesions play a critical role in prognosis. These injuries, reported in up to 30–70% of cases depending on the diagnostic strategy, are frequently underdiagnosed and may lead to chronic instability, stiffness, and poor functional outcomes [11–13]. Although intramedullary nailing combined with early rehabilitation is generally associated with improved healing and functional recovery, this approach is not always feasible in polytrauma patients, particularly in the presence of severe soft-tissue damage or vascular injury [1, 14]. As a result, treatment strategies remain heterogeneous, and outcomes vary across studies.

Despite growing interest in this injury pattern, the available literature is mainly composed of small retrospective series with limited follow-up. Most studies focus on isolated aspects of the injury, such as fracture healing, ligamentous lesions, or functional outcomes, but rarely provide a comprehensive analysis integrating all the parameters. Moreover, the prognostic impact of associated meniscoligamentous injuries remains insufficiently characterized.

Therefore, the primary objective of this study was to identify variables affecting bone union and risk factors for nonunion. The secondary objective was to evaluate the impact of articular involvement, meniscoligamentous lesions and postoperative complications on clinical, radiological and functional outcomes.

## Materials and methods

### Population

This retrospective bicentric cohort included patients with floating knee between January 2008 and December 2021.

The initial database included 105 floating knees in 101 patients.

Exclusion criteria were associated limb injuries likely to dominate functional prognosis, patterns not fitting the Ran classification, age <16 or >65 years, periprosthetic, physeal, recurrent or pathological fractures.

**Figure 2:**
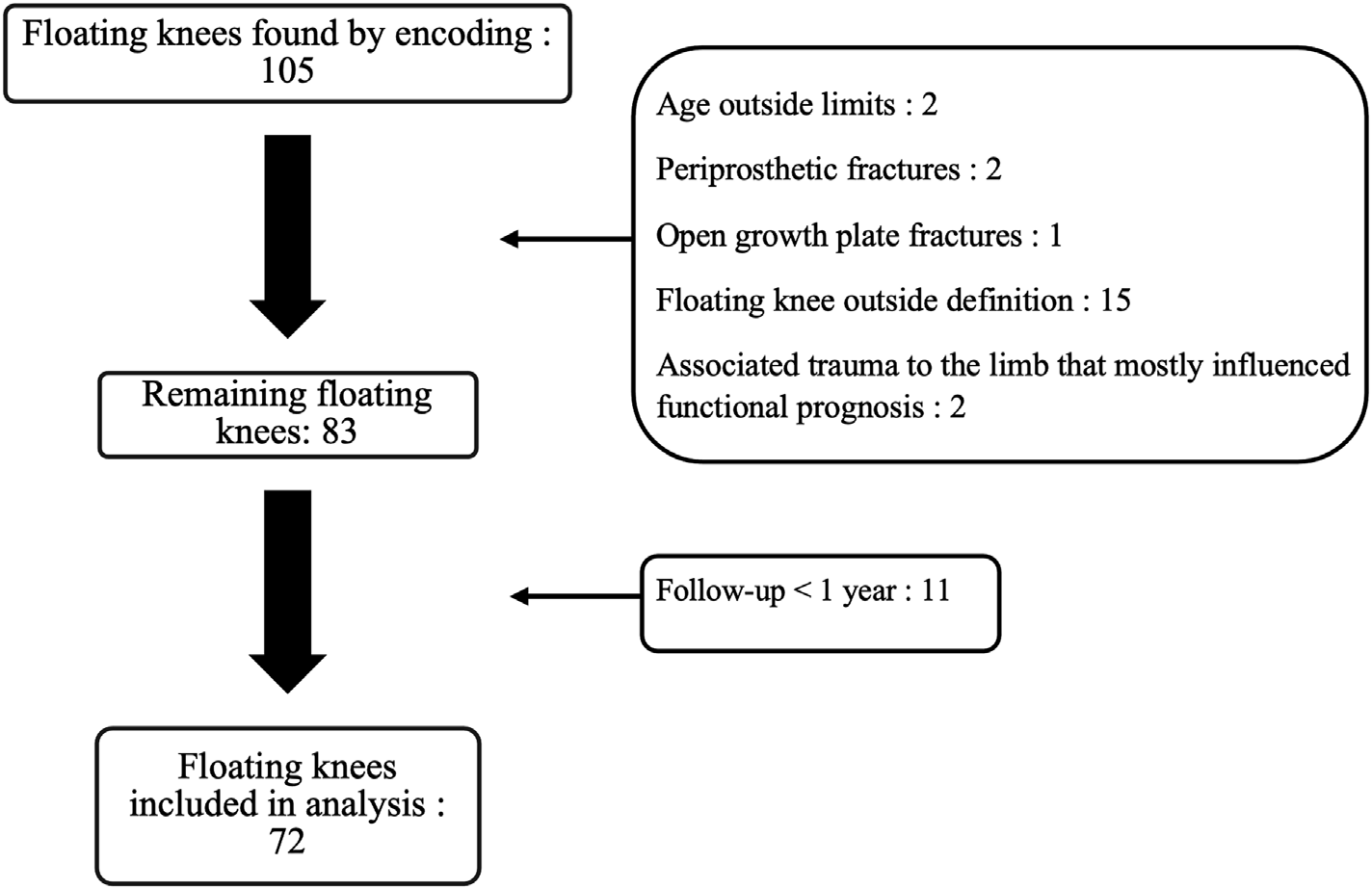
Flowchart.

After exclusion, 72 floating knees in 70 patients were included. 40 (60%) were treated at the Edouard Herriot Hospital and 29 (40%) at the Lyon Sud Hospital Center (Figure 2). There was 58 men and 12 women, with a sex ratio of 4.8:1. Mean follow-up was 53.9 months [9–188]. Mean age at time of injury was 34 years [16–62]. In this young population, 50 (71.4%) patients had no medical background. 26 (37.1%) were active smokers.

### Injury characteristics

Fractures were classified according to Ran [9] and Gustilo and Anderson [15]. Neurological, vascular, meniscoligamentous and associated injuries were studied.

Postoperatively, there was no standardized rehabilitation protocol.

Ran type 1 was the most frequent pattern (Table 1). Patellar fractures (Ran 3 type) were present in 13 cases (18.1%). Comminution was present in 12 (16.7%) femoral fractures and 21 (29.2 %) tibial fractures. Segmental fractures were observed in 1 (1.4%) femur and 6 (8.3%) tibias. Open fractures involved femur in 33 cases (45.8%), tibia in 36 (50%) and patella in 8 (61.5%). At least one fracture was open in 50 floating knees (69.4%).

**Table 1:** Distribution of floating knees according to Ran's classification and skin condition (Gustilo and Anderson classification).

Classifications	Subtypes	Floating knee *n* (%)	Femur *n* (%)	Tibia *n* (%)	Patella *n* (%)
Ran 1 (extra-articular)	–	35 (48.6%)	–	–	–
Ran 2 (intra-articular)	2a	13 (18.1%)	–	–	–
	2b	11 (15.3%)	–	–	–
Ran 3 (intra-articular + patella)	3a	3 (4.2%)	–	–	–
	3b	10 (13.9%)	–	–	–
Comminution			12 (16.7 %)	21 (29.2 %)	10 (13.9%)
Segmental			1 (1.4 %)	6 (8.3 %)	–
Closed fracture	–	–	39 (54.2%)	36 (50%)	5 (38.5%)
Open fracture	–	–	33 (45.8%)	36 (50%)	8 (61.5%)
Gustilo I	–	–	3 (4.2%)	6 (8.3%)	/
Gustilo II	–	–	24 (33.3%)	16 (22.2%)	8 (61.5%)
Gustilo III	IIIA	–	4 (5.6%)	4 (5.6%)	/
	IIIB	–	2 (2.8%)	5 (6.9%)	/
	IIIC	–	/	5 (6.9%)	/

Initial surgical management commonly involved damage-control strategies, with 29 (40.3%) femoral external fixations and 36 (50%) tibial external fixations. Following initial stabilization, 26 femoral fractures (36.1%) and 39 tibial fractures (54.2%) underwent definitive osteosynthesis. In Ran type 3 knees, 7 patellar fractures (53.8%) were managed non-operatively, 5 (38.5%) underwent fixation, and 1 (7.7%) underwent patellectomy.

Average delay between initial and definitive surgery was 16.4 [1–60] days for femur and 38.4 [3–210] days for tibia. The average time to surgical management was 15.7 days [2–84] for patella.

9 (12.5%) associated arterial injuries were found. 4 (44.4%) required emergent surgical repair. Neurological injuries were present in 12 cases (16.7%), mostly involving the common fibular nerve. 17 (23.6%) associated osteoarticular injuries of the limb were recorded.

Only 13 (18.6%) patients had an isolated floating knee. The remaining 57 (81.4%) patients were polytraumatized.

There were 23 (31.9%) cases of meniscoligamentous injuries. 9 (39.1%) were single ligament injuries. 8 (34.8%) were multiligament injuries. There were 2 (8.7%) isolated meniscal injuries, 2 (8.7%) patellar tendon avulsions, and 2 (8.7%) meniscoligamentous injuries. Mean diagnosis delay was 11.2 months [0–167]. Diagnosis methods included 10 clinical exams (43.5%), 6 intraoperative findings (26.1%), 5 MRI (21.7%), 1 CT (4.3%) and 1 radiograph (4.3%). 8 injuries (34.8%) were surgically treated at a mean of 29.2 days post-injury, including two treated concomitantly with fracture fixation.

Meniscoligamentous lesions were significantly associated with increasing Ran stage (Cochran–Armitage, *p* < 0.05) and with chronic instability (Fisher, *p* < 0.05), but not with postoperative stiffness (*p* = 0.71).

### Bone healing

Union was achieved when 3 out of 4 cortices were fused radiographically.

Nonunion was defined as the absence of radiographic healing at six months, based on incomplete cortical bridging (fewer than 3 out of 4 cortices) [7, 16]. It could be septic or mechanical.

### Complications

Muscular, cutaneous, septic, neurological, osteoarticular, and general complications were recorded. Treatment failure was defined as inability to preserve the native knee.

### Clinical, radiological and functional outcomes

Clinical and radiographic examination of the knee at the last follow-up was recorded.

Functional parameters were assessed as well as the Karlström and Olerud score (KOS) [17].

### Statistical analysis

The analyses were performed using GPL RStudio 1.0 software (Posit.Software, PBC). Descriptive statistics summarized the characteristics of the groups and subgroups.

Quantitative data were expressed as means if they followed a normal distribution, otherwise as medians.

For nominal qualitative variables, Fisher's test was used to analyze associations. When the analysis involved an increasing nominal variable or an ordinal variable, Cochran–Armitage's trend test was used. When both variables were ordinal, the association was evaluated using Kendall's tau-b coefficient. For sample sizes of less than 5, the results were verified by a fixed-margin permutation test for the Cochran–Armitage test and/or tau-b. For binary outcomes based on quantitative variables, logistic regressions were used.

The significance threshold was *p* = 0.05.

## Results

### Bone healing

Mean time union was 10.5 [2–84] months for femur, 7.5 months [2–24] for tibia, and 4.4 months [1–9] for patella.

There were 49 (68.1%) femoral nonunions including 4 septic and 45 mechanical, compared with 41 (56.9%) in tibia, including 11 septic and 30 mechanical. Patellar healing was complicated by 5 mechanical nonunions (41.7%). 33 (45.8%) patients developed nonunion of the femur and tibia. Only 14 floating knees (19.4%) healed without nonunion. Overall, 56 knees (77.8%) developed at least one nonunion.

Significant predictors of nonunion included open fractures of the femur and tibia and tibial vascular injury. No significant predictor was identified for patellar nonunion (Table 2).

**Table 2:** Risk factors for nonunion.

Factor investigated	Femoral nonunion	Tibial nonunion
Joint fracture (Ran 2a, 2b, 3a, 3b)	No	No
Open fracture	Yes (*p* = 0.01)	Yes (*p* = 0.03)
Comminution	No	No
Segmental fracture	No	No
Vascular lesions	No	Yes (*p* = 0.001)
Meniscoligamentous injury	No	No
Exofixation	No	No
Type of osteosynthesis	No	No
Age	No	No
Active smoking	No	No

### Complications

46 (56.9%) floating knees developed at least one complication.

4 (5.6%) acute compartment syndromes occurred.

12 (16.7%) cases presented with cutaneous complications, 11 (29.3%) requiring reconstruction surgery.

Surgical site infection occurred in 22 knees (30.6%), diagnosed at a mean of 22.5 months (1–78). Infection was significantly associated with open fractures of the femur (*p* < 0.001) and tibia (*p* < 0.001) at Fisher’s exact test.

31 (43.1%) floating knees had at least one osteoarticular or material-related complication. 10 (13.9%) developed stiffness (<90° flexion at 3 months). No significant association was found between Ran classification and osteoarticular complications.

6 systemic complications were recorded, including 2 (2.8%) fat embolisms and 4 (5.6%) venous thromboembolic events. No death occurred.

7 (9.7%) knees could not be saved. Among these, there were 2 (2.8%) femorotibial arthrodeses, 3 (4.2%) resection prothesis and 2 (2.8%) amputations. All involved open and articular fractures.

### Follow-up outcomes

Clinical data were available for 66 patients. 28 (42.2%) had a normal range of motion. 11 (15.3%) had instability.

Radiographically, 48.6% developed knee osteoarthritis at the last follow-up. The incidence of osteoarthritis increased significantly with Ran stage (*p* < 0.001).

KOS was available for 67 patients. Most patients had poor scores (20 patients, 29.9%), 14 (20.9%) were acceptable, 17 (25.4%) were good and 16 (23.9%) were excellent. Predictors of poor functional outcome included meniscoligamentous injuries (*p* < 0.05), open fracture (*p* < 0.001), infection (*p* < 0.001), and higher Ran stage (Kendall tau-b, *p* < 0.001).

## Discussion

### Main findings

Nonunion rates were particularly high (68.1% femur, 56.9% tibia, 41.7% patella), with open fractures and tibial vascular injuries as the main predictors. Mean union time was 10.5 months for the femur, 7.5 months for the tibia, and 4.4 months for the patella.

For meniscoligamentous injuries (31.9%), the mean time to diagnosis was 11.2 months. Their frequency increased significantly with fracture severity. Functionally, patients with this type of injury had significantly poorer KOS results and more chronic instability.

The frequency of osteoarthritis increased significantly with stages of Ran classification, showing a prognostic impact of articular and comminuted injuries and patellar involvement.

Infections (30.6%) were significantly more frequent in open fractures.

Overall functional outcomes were mostly poor. A significant correlation was found between poor outcomes and severity according to Ran, open fractures, meniscoligamentous injuries and infection.

### Comparison with the literature

Our nonunion rates were higher than those reported in the literature, which range from 4% to 28% for the femur and from 3% to 36% for the tibia [2–6]. This discrepancy likely reflects the severity of injury patterns in our cohort, characterized by a high proportion of comminuted, articular and open fractures (Table 1). In addition to fracture severity and soft-tissue damage, the frequent use of staged fixation strategies within a damage control framework may have contributed to these results [14, 18]. Although often necessary in polytrauma patients, this approach may lead to delayed definitive fixation and suboptimal mechanical conditions for bone healing. Furthermore, the presence of associated vascular injuries, particularly affecting the tibia, may have further compromised the local biological environment.

Among studies, reported healing times also vary widely, depending on fracture complexity and treatment strategies. Shorter times are generally observed in selected populations treated with intramedullary nailing [1, 19] (Table 3), typically involving younger patients and excluding more complex fracture patterns as Ran types 2 and 3, which have consistently been associated with prolonged healing in several series [2, 4, 20, 21].

**Table 3:** Comparison with literature.

Author, year of publication	Number of cases	Classification	Average follow-up (months)	Nonunion rate (%)	Time to union (months)	Meniscoligamentous injury (%)	Infectio*n* (%)	Karlström & Olerud’s score
Ostrum [1], 2000	20	Winquist	18.5	/	Femur: 3.6	/	5%	88% good or excellent
					Tibia: 5.7			
								6% acceptable
								6% poor
Rethnam [13], 2009	29	Blake and Mc Bryde	24	/	Femur:	14%	/	52% excellent
					-extra articular : 4.8			34% good
								10% acceptable
					-intra articular : 5.6			3% poor
					Tibia :			
					-extra articular : 5.2			
					-intra articular : 4.2			
Hegazy [4], 2011	15	Fraser	23.5	/	Femur : 3.7	/	7%	53% excellent
					Tibia : 5.8			27% good
								13% acceptable
								1% poor
Feron [16], 2015	172	Fraser	34	Total : 21%	/	6%	/	17% excellent
								37% good
								24% acceptable
								12,5% poor
Shahzad [8], 2015	65	Fraser	6	Femur : 11%	/	/	Femur : 17%	25% excellent
				Tibia : 14%			Tibia : 20%	40% good
				Total : 12%				25% acceptable
								11% poor
Kulkarni [7], 2018	90	Fraser, Ran	36	Femur : 27%	Femur : 10.5	/	11%	24% excellent
				Tibia : 32%	Tibia : 9.5			29% good
								27% acceptable
								12% poor
Ethiraj [18], 2022	46	Ran	12	/	Femur :	/	22%	DCO : 15% excellent
					-DCO : 11			
					-ETC : 9			40% good
					Tibia :			35% acceptable
					-DCO : 10			10% poor
					-ETC : 9			
								ETC:
								29% excellent
								62% good
								9,5% acceptable
								0 poor
Meccariello [2], 2024	168	Fraser, Ran	89	Femur : 22%	/	79%	7%	29% excellent
				Tibia : 65%				35% good
				Femur & tibia : 13%				23% acceptable
								14% poor
Our study	72	Ran	54	Femur : 68%	Femur : 10.5	32%	31%	24% excellent
								25% good
								21% acceptable
				Tibia : 57%	Tibia : 7.5			30% poor
				Femur & tibia : 46%				

DCO: damage control orthopedics; ETC : early total care.

Differences in reported healing times across studies may also reflect variability in the assessment of bone union and lack of standardized definition of nonunion across studies. We defined nonunion as the absence of radiographic healing at six months, based on the criterion of incomplete cortical bridging, which is consistent with definitions used in previous studies [7, 16]. However, other authors rely on the absence of radiological progression over time, or use longer postoperative thresholds up to 9 months, before establishing the diagnosis [5]. This heterogeneity in definitions should be taken into account when comparing results across studies.

Regarding meniscoligamentous injuries, delayed diagnosis is known to be responsible for poor functional outcomes [22]. Liu et al. [12] systematically performed knee testing after osteosynthesis as well as diagnostic arthroscopy. Such an invasive approach resulted in high reported lesion rate (70%), with likely overdiagnosis, and the functional impact was not studied. This approach is favored by many authors instead of preoperative MRI, which is often not available or inappropriate in a damage-control context. It also avoids postoperative MRI, which is often limited by osteosynthesis artefacts [13, 23].

Meccariello et al. [2] used the same approach and reported 78.6% meniscoligamentous injuries. They studied the predictive value of a new prognostic classification that included meniscoligamentous injuries, and concluded that the KOS was better correlated with this classification than with Ran classification.

Articular involvement in fractures is usually associated with stiffness. Although this association was not significant in our cohort, it has been reported by Kulkarni et al. and Kurkowski et al., who also identified tibial open fractures, extensor mechanism injuries and reoperations as risk factors [7, 24]. The impact of patellar fractures in our results could not be demonstrated due to lack of statistical power and has only been studied in a few series [2, 9, 24]. Ran et al. [9] reported an incidence of 25% patellar fractures, of which 29% developed stiffness. The classification proposed by Meccariello et al. [2] was statistically predictive of a worsened KOS according to the different stages.

Regarding surgical site infections, their incidence in our cohort aligns with the upper range of published data [5, 8].

Regarding functional outcomes, many studies reported mainly good to excellent results [5, 16] (Table 3). The high rates of nonunion and infection in our series, as well as the absence of systematic evaluation of meniscoligamentous injuries, may partly explain our results. The very elevated proportion of Ran type 1 floating knees in some studies [5, 16] can also explain their better functional scores.

### Strengths and limitations

The strength of our study lies in its sample size. Although it is not the largest series published, it allows for subgroup analyses, some of which yielded significant results using statistical analyses adapted to small sample sizes. The average follow-up period of 53.9 months, or approximately 4.5 years, is one of the longest found in the literature [3, 5, 6, 25], but does not allow for the evaluation of long-term post-traumatic osteoarthritis.

This study has several limitations that should be acknowledged. First, its retrospective design inherently exposes it to selection bias, information bias related to incomplete or missing data, and potential confounding factors, with no possibility of randomization or control over treatment allocation. Second, although relatively large for such a rare condition, the sample size may still be insufficient to provide adequate statistical power for all subgroup analyses.

In addition, the absence of a standardized rehabilitation protocol across patients may have influenced both bone healing and functional outcomes. The bicentric design, involving variability in surgical strategies, timing of definitive fixation, and postoperative management, as well as the multiplicity of surgeons, further contributed to heterogeneity in patient care. Moreover, the long inclusion period, spanning more than a decade, likely reflects changes in surgical techniques, implants, and overall trauma management, which may have impacted the results.

Finally, the heterogeneity in injury patterns, particularly the high proportion of open and severe fractures, may limit the generalizability of our findings and partly explain the high rates of nonunion and complications observed in this cohort.

In line with recent publications, the study of extensor apparatus injuries, systematic screening for meniscoligamentous lesions, and the relevance of rehabilitation protocols must be further analyzed [2, 24].

## Conclusion

This study highlights the importance of early and meticulous management of skin and vascular lesions to limit their impact on bone union. Skin opening and articular injuries make debridement and anatomical reduction essential. Meniscoligamentous injuries must be identified early in the management process. Follow-up must be prolonged to detect instability, stiffness, and knee osteoarthritis. Functional outcomes depend on multiple parameters whose prognostic value has been demonstrated.

## Data Availability

Data originate from the Hospices Civils de Lyon medical information systems and cannot be publicly released for ethical reasons.

## References

[1] Ostrum RF (2000) Treatment of floating knee injuries through a single percutaneous approach. Clin Orthop Relat Res 375, 43–50. 10.1097/00003086-200006000-00006 10853152

[2] Meccariello L, Pica R, Erasmo R, Ronga M, Ippolito F, Vicenti G, et al. (2024) Floating knee : a new prognostic classification. Injury 55, 111471. 39542575 10.1016/j.injury.2024.111471

[3] Nouraei MH, Hosseini A, Zarezadeh A, Zahiri M (2013) Floating knee injuries : results of treatment and outcomes. J Res Med Sci 18, 1087–1091. 24523801 PMC3908531

[4] Hegazy AM (2011) Surgical management of ipsilateral fracture of the femur and tibia in adults (the floating knee): postoperative clinical, radiological, and functional outcomes. Clin Orthop Surg 3, 133–139. 21629474 10.4055/cios.2011.3.2.133 PMC3095784

[5] Hee HT, Wong HP, Low YP, Myers L (2001) Predictors of outcome of floating knee injuries in adults: 89 patients followed for 2-12 years. Acta Orthop Scand 72, 385–394. 11580128 10.1080/000164701753542050

[6] Kao FC, Tu YK, Hsu KY, Su JY, Yen CY, Chou MC (2010) Floating knee injuries: a high complication rate. Orthopedics 33, 14–18. 10.3928/01477447-20091124-04 20055342

[7] Kulkarni MS, Aroor MN, Vijayan S, Shetty S, Tripathy SK, Rao SK (2018) Variables affecting functional outcome in floating knee injuries. Injury 49, 1594–1601. 29885963 10.1016/j.injury.2018.05.019

[8] Shahzad K, Khan RDA, Yasin A (2015) Floating knee injuries: postoperative complications and outcome. J Pak Med Assoc 65, 195–201. 26878519

[9] Ran T, Hua X, Zhenyu Z, Yue L, Youhua W, Yi C, et al. (2013) Floating knee: a modified Fraser’s classification and the results of a series of 28 cases. Injury 44, 1033–1042. 23312566 10.1016/j.injury.2012.12.012

[10] Paul GR, Sawka MW, Whitelaw GP (1990) Fractures of the ipsilateral femur and tibia: emphasis on intra-articular and soft tissue injury. J Orthop Trauma 4, 309–314. 2231130 10.1097/00005131-199004030-00013

[11] Szalay MJ, Hosking R, Annear P (1990) Injury of knee ligament associated with ipsilateral femoral shaft fractures and with ipsilateral femoral and tibial shaft fractures. Injury 21, 398–400. 2276807 10.1016/0020-1383(90)90129-i

[12] Liu Y, Zhang J, Zhang S, Li R, Yue X (2015) Concomitant ligamentous and meniscal injuries in floating knee. J Orthop Traumatol 16, 1168–1172. PMC4358564 25785109

[13] Rethnam U, Yesupalan RS, Nair R (2009) Impact of associated injuries in the floating knee : a retrospective study. BMC Musculoskelet Disord 10, 7. 19144197 10.1186/1471-2474-10-7 PMC2630294

[14] Bertrand ML, Andrés-Cano P (2015) Management of the floating knee in polytrauma patients. Open Orthop J 9, 347–355. 26312119 10.2174/1874325001509010347 PMC4541470

[15] Gustilo RB, Anderson JT (1976) Prevention of infection in the treatment of one thousand and twenty-five open fractures of long bones : retrospective and prospective analyses. J Bone Joint Surg Am 58, 453–458. 773941

[16] Feron JM, Bonnevialle P, Pietu G, Jacquot F (2015) Traumatic floating knee: a review of a multi-centric series of 172 cases in adults. Open Orthop J 9, 356–360. 10.2174/1874325001509010356 PMC4541414 26312122

[17] Karlström G, Olerud S (1977) Ipsilateral fracture of the femur and tibia. J Bone Joint Surg Am 59, 240–243. 845210

[18] Ethiraj P, Shringeri AS, Prasad PA, Shanthappa AH, Nagarajan V (2022) Early total care versus damage control orthopedics in floating knee injury : analysis of radiological and functional outcomes. Cureus 14(6), e25615. 35784973 10.7759/cureus.25615 PMC9249040

[19] Behr JT, Apel DM, Pinzur MS, Dobozi WR, Behr MJ (1987) Flexible intramedullary nails for ipsilateral femoral and tibial fractures. J Trauma 27, 1354–1357. 3694726 10.1097/00005373-198712000-00006

[20] Vallier HA, Manzano GW (2020) Management of the floating knee: ipsilateral fractures of the femur and tibia. J Am Acad Orthop Surg 28, e47–e54. 31305352 10.5435/JAAOS-D-18-00740

[21] Hung S, Lu Y, Huang H, Lin Y, Chang J, Chen J, et al. (2007) Surgical treatment of type II floating knee : comparisons of the results of type IIA and type IIB floating knee. Knee Surg Sports Traumatol Arthrosc 15, 578–586. 17203298 10.1007/s00167-006-0252-1

[22] Andrade-Silva FB, Carvalho A, Mansano C, Giese A, De Camargo Leonhardt M, Barbosa D, et al. (2017) Functional results and isokinetic muscle strength in patients with Fraser type I floating knee treated with internal fixation. Injury 48, S2–S5. 10.1016/S0020-1383(17)30767-2 29145963

[23] Muñoz Vives J, Bel JC, Capel Agundez A, Chana Rodríguez F, Palomo Traver J, Schultz-Larsen M, et al. (2016) The floating knee: a review on ipsilateral femoral and tibial fractures. EFORT Open Rev 1, 375–382. 28461916 10.1302/2058-5241.1.000042 PMC5367526

[24] Kurkowski S, Catlett S, Gerak S, Mor Huertas A, Beltran M (2025) Articular involvement impacts unplanned reoperation rates in floating knee injuries. Injury 56, 112679. 40816063 10.1016/j.injury.2025.112679

[25] Dwyer AJ, Paul R, Mam MK, Kumar A, Gosselin RA (2005) Floating knee injuries : long-term results of four treatment methods. Int Orthop 29, 314–318. 16132984 10.1007/s00264-005-0679-x PMC3456641

